# FAN1 controls mismatch repair complex assembly via MLH1 retention to stabilize CAG repeat expansion in Huntington’s disease

**DOI:** 10.1016/j.celrep.2021.109649

**Published:** 2021-08-31

**Authors:** Robert Goold, Joseph Hamilton, Thomas Menneteau, Michael Flower, Emma L. Bunting, Sarah G. Aldous, Antonio Porro, José R. Vicente, Nicholas D. Allen, Hilary Wilkinson, Gillian P. Bates, Alessandro A. Sartori, Konstantinos Thalassinos, Gabriel Balmus, Sarah J. Tabrizi

**Affiliations:** 1 UCL Huntington’s Disease Centre, Department of Neurodegenerative Disease, UCL Queen Square Institute of Neurology, Queen Square, London WC1N 3BG, UK; 2 UK Dementia Research Institute, University College London, London WC1N 3BG, UK; 3 Institute of Structural and Molecular Biology, Division of Biosciences, University College London, London WC1E 6BT, UK; 4 Institute of Molecular Cancer Research, University of Zurich, Zurich 8057, Switzerland; 5 UK Dementia Research Institute, University of Cambridge, Cambridge CB2 0AH, UK; 6 School of Biosciences, Cardiff University, Cardiff CF10 3AX, UK; 7 CHDI Management/CHDI Foundation, Princeton, NJ 08540, USA; 8 Institute of Structural and Molecular Biology, Birkbeck College, University of London, London WC1E 7HX, UK; 9 Department of Clinical Neurosciences, University of Cambridge, Cambridge CB2 0AH, UK

**Keywords:** Huntington’s disease, repeat expansion, GWAS, CAG instability, DNA repair, mismatch repair, FAN1, MLH1, MSH3, FAN1 nuclease activity

## Abstract

CAG repeat expansion in the *HTT* gene drives Huntington’s disease (HD) pathogenesis and is modulated by DNA damage repair pathways. In this context, the interaction between FAN1, a DNA-structure-specific nuclease, and MLH1, member of the DNA mismatch repair pathway (MMR), is not defined. Here, we identify a highly conserved SPYF motif at the N terminus of FAN1 that binds to MLH1. Our data support a model where FAN1 has two distinct functions to stabilize CAG repeats. On one hand, it binds MLH1 to restrict its recruitment by MSH3, thus inhibiting the assembly of a functional MMR complex that would otherwise promote CAG repeat expansion. On the other hand, it promotes accurate repair via its nuclease activity. These data highlight a potential avenue for HD therapeutics in attenuating somatic expansion.

## Highlights

• FAN1 binds MLH1 via conserved ^126^SPYF^129^ residues, acting as a canonical MIP-box
• FAN1-MLH1 binding regulates mismatch repair activity and complex formation
• FAN1-MLH1 binding regulates the *HTT* CAG expansion rate

## Graphical abstract

**Figure:**
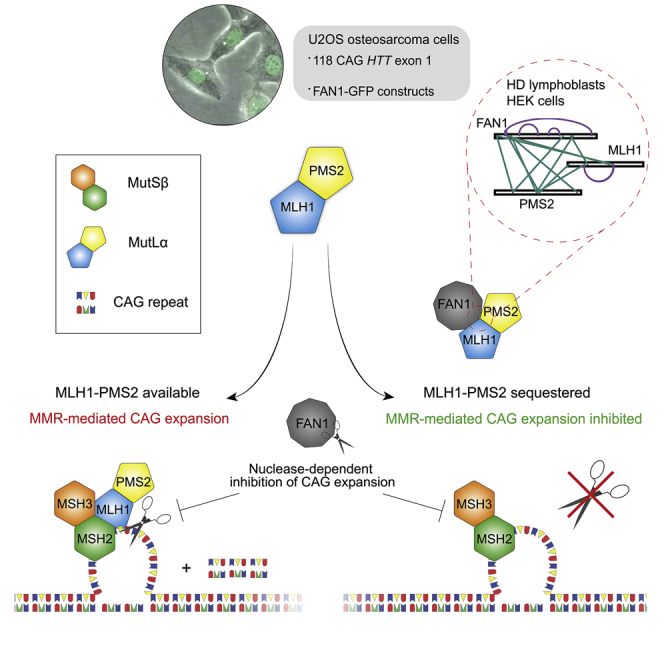

## Teaser

FAN1 modifies Huntington’s disease pathogenesis, but the mechanism has remained elusive. Goold et al. demonstrate that FAN1 binds MLH1 through residues ^126^SPYF^129^, competing with MSH3, and sequesters MLH1 from the mismatch repair pathway. In turn, this reduces mismatch repair activity and suppresses expansion of the pathogenic *HTT* CAG trinucleotide repeat.

## Introduction

Huntington’s disease (HD) is a monogenic neurodegenerative condition arising due to inheritance of ≥36 CAG repeats in exon 1 of the huntingtin (*HTT*) gene. Expansion of CAG repeats occurs in selected somatic and selected meiotic tissues, but the neurodegeneration is primarily due to loss of neurons in the striatum and cortex (MacDonald et al., 1993; Pinto et al., 2013; Rikitake et al., 2020; Tomé et al., 2013). Faster somatic expansion rates correlate with earlier age at onset and faster disease progression (Bates et al., 2015; Rawlins et al., 2016; Flower et al., 2019; Swami et al., 2009; Wright et al., 2019). The expanded CAG repeat may be pathogenic through several mechanisms, including at the protein level through translation into a longer, more toxic polyglutamine tract; at the RNA level through the incomplete splicing of *HTT* (Neueder et al., 2017; Sathasivam et al., 2013), RAN translation, or RNA secondary structure (Bañez-Coronel et al., 2015, Schilling et al., 2016); and at the DNA level through an effect on transcription and DNA repair activity (Wright et al., 2020). Targeting repeat expansion, the most proximal pathogenic event, represents a prime therapeutic opportunity in HD and potentially other trinucleotide disorders (Tabrizi et al., 2020). In recent years, several genome-wide association studies (GWASs) have identified DNA repair genes as main modifiers of HD onset and progression (GeM-HD Consortium, 2019). The strongest signal comes from genetic variation in the DNA repair gene *FAN1*, a nuclease of the Fanconi anemia (FA) pathway (MacKay et al., 2010; Smogorzewska et al., 2010), while other prominent modifications are in *MSH3*, *MLH1*, and *PMS2*, members of the mismatch repair (MMR) pathway (Jiricny, 2006). Similarly, transcriptome-wide association studies (TWASs) show a signature in which reduced *MSH3* but increased *FAN1* expression are associated with later onset, slower progression, and CAG repeat stability (Flower et al., 2019; Goold et al., 2019). We and others have demonstrated in cell and animal models that deficiency of MSH3, MSH2, MLH3, PMS2, and MLH1 or increased expression of FAN1 (Tomé et al., 2013; Pinto et al., 2013; Miller et al., 2020) prevents somatic expansion. This is consistent with analyses linking *FAN1* loss-of-function variants, such as p.R507H (GeM-HD Consortium, 2019), with earlier onset. Therefore, in the context of HD, FAN1 expression has a dose-dependent protective effect on CAG repeat expansion, providing a credible mechanism for its defensive influence *in vivo*.

Despite this, the molecular relationship between MMR and FAN1 is not well understood. MMR relies on the MutSβ heterodimer (MSH3-MSH2) to recognize large loops in slipped DNA and to recruit MutLα (MLH1-PMS2) to incise DNA through its endonuclease activity. Thereafter, repair is conducted by a DNA polymerase and ligase 1 (LIG1), incorporating additional CAG repeat units. On the other hand, FAN1 is an endonuclease and 5′–3′ exonuclease that excises aberrant interstrand crosslinks (ICLs) that impair transcription and ensures the recovery of stalled replication forks (Huang and D’Andrea, 2010; Lachaud et al., 2016; Chaudhury et al., 2014). How FAN1 protects against CAG repeat instability remains unclear, with some data suggesting FAN1’s DNA-binding capacity may be important (Kim et al., 2020). Interestingly, FAN1 interacts directly with MLH1 (Pinto et al., 2013; Rikitake et al., 2020; Tomé et al., 2013) but, to our knowledge, the nature and purpose of this interaction has not been explored in a HD context. Recent evidence in a HD mouse model supports the protective effect of FAN1 at CAG repeats and shows that it acts through MLH1 (Loupe et al., 2020). These physical and genetic links prompted us to further investigate the mechanistic significance of the FAN1-MLH1 relationship.

Using the U2OS cell line, well established in the FAN1 field (MacKay et al., 2010; Munoz et al., 2014), we show that an evolutionary conserved functional domain of FAN1 (^126^SPYF^129^) is responsible for binding MLH1 and is important for CAG repeat stability. Additionally, we highlight the *in vivo* relevance of this interaction by demonstrating that FAN1 binds MLH1 in multiple human and mouse HD models. The FAN1-MutL interaction prevents the recruitment of MLH1 to the MutSβ complex, thereby reducing somatic expansion. We further show that FAN1’s nuclease activity plays an active role in suppressing expansion. Therefore, promoting the FAN1-MutL complex interaction represents an unexplored therapeutic strategy in HD and potentially other trinucleotide disorders.

## Results

### FAN1-MLH1 binding demonstrated *in vitro and in vivo* in multiple HD models

**Figure 1:**
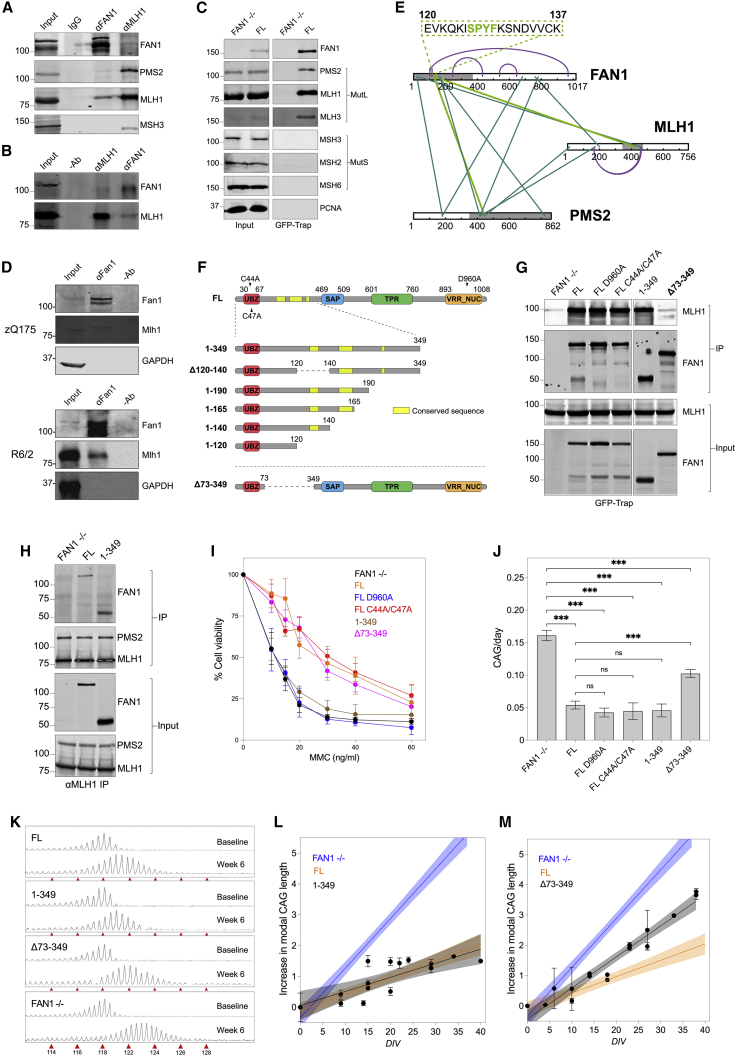
The FAN1 N-terminal region (p.73-349) mediates its interaction with MLH1 and its effect on CAG stabilization activity (A) CoIP extracts from human HD iPSCs showing FAN1 interacts with MutLα components MLH1 and PMS2. Note that MSH3 is absent from the anti-FAN1 IP fraction (n = 3 biological replicates). (B) CoIP extracts from human HD lymphoblasts confirming FAN1 interacts with MLH1 (n = 3 biological replicates). (C) Pull-down assays using GFP-Trap beads in U2OS cells showing FAN1 interacts with MutL components, but not MutS components or PCNA (proliferating cell nuclear antigen). FAN1^−/−^ cells act as a negative control, demonstrating specificity of the pull-down (n = 4 biological replicates). (D) CoIP of cortical extracts from mouse zQ175 at 6 months of age confirming FAN1 interacts with MLH1. Observations were also confirmed in R6/2 HD mice at 12 weeks of age (zQ175, n = 3 biological replicates; R6/2, n = 2 biological replicates). (E) Crosslinks identified between FAN1, MLH1, and PMS2 in unstimulated HEK293T cells and HD lymphoblasts. Grey parts on the proteins are structurally unsolved (no PDB structure available). Turquoise line, interprotein; purple line, intraprotein; green line, crosslinks close to the SPYF motif. See also Figure S1A and Table S1. (F) Schematic illustrating FAN1 constructs cloned into U2OS system. Locations of UBZ null (C44A/C47A) and nuclease null (D960A) mutations are also outlined. UBZ, ubiquitin-binding zinc-finger domain; SAP, SAF-A/B, Acinus and PIAS domain; TPR, tetratricopeptide repeat domain; VRR_NUC, virus-type replication-repair nuclease domain. (G) Pull-down using GFP-Trap beads in U2OS cells expressing GFP-FAN1 deletion constructs. FAN1^Δ73–349^ (highlighted in bold) did not interact with MLH1. Note that inactivation of UBZ or VRR_NUC domains (C44A/C47A and D960A mutants, respectively) does not affect FAN1-MLH1 interaction (n = 3 biological replicates). (H) CoIP extracts using αMLH1 antibody in U2OS cells showing N-terminal FAN1^1–349^ is sufficient to interact with MLH1 (n = 4 biological replicates). (I) MMC viability curves in U2OS cells expressing FAN1 variants (mean ± SD) showing lower viability when FAN1 lacks an intact nuclease domain (n = 5–8 biological replicates; n = 3 technical replicates). See also Figure S1E. (J) CAG expansion rates in U2OS cells expressing truncated FAN1 constructs, including mutations within key functional domains (UBZ, C44A/C47A; VRR_NUC, D960A). Note that only FAN1^Δ73–349^ shows a higher expansion rate than cells expressing FAN1^FL^ but does not equate to FAN1^−/−^ (mean ± SEM, n = 2–5 biological replicates, n = 3–6 technical replicates, F(5,97) = 40.8, p < 0.001 by one-way ANOVA with false discovery rate [FDR] correction of 5%). ^∗∗∗^p < 0.001; ns, non-significant. (K–M) Fragment analysis traces illustrating expansion of the exogenous *HTT* 118 CAG repeat in U2OS cells expressing FAN1 constructs over 6 weeks in culture (K) with time courses plotted (L and M). Note that cells expressing FAN1^Δ73–349^ (individual data points shown) expand at a rate between that of FAN1^FL^ or FAN1^1–349^ (individual data points shown) and FAN1^−/−^ cells (mean ± SD, 95% confidence interval [CI] in shaded areas, n = 2–5 biological replicates, n = 3–6 technical replicates).

Because of the strong genetic evidence linking FAN1 and MMR proteins in the pathogenesis of HD, we speculated that FAN1 could directly interact with MMR factors at CAG repeats to modulate expansion. To test the functional significance of the FAN1-MLH1 interaction in an HD context, we first tested this hypothesis in induced pluripotent stem cells (iPSCs) derived from a juvenile HD patient originally carrying 125 CAGs. Immunoprecipitation (IP) using FAN1 antibodies showed MLH1 and PMS2 were present in FAN1 pull-down fractions, whereas MSH3 was absent, and conversely, FAN1 was present in MLH1 pull-down fractions alongside PMS2 and MSH3 (Figure 1A). To confirm this interaction in independent cell lines, we used HD lymphoblastoid (LB) cells carrying more typical, shorter, disease-associated repeat lengths (Figure 1B). To exclude antibody-specific artifacts, we validated this interaction in U2OS cells expressing GFP-FAN1 and confirmed that MLH1, PMS2, and MLH3 can be detected in GFP-Trap pull-down fractions, whereas MSH2, MSH3, and MSH6 were absent (Figure 1C). Finally, to demonstrate the significance of this interaction *in vivo*, we showed that FAN1 and MLH1 interact in cortical extracts of zQ175 and R6/2 HD mice (Figure 1D).

To further dissect the interaction between FAN1 and the MLH1-PMS2 heterodimer, we performed crosslinking IP mass spectrometry (xIP-MS) experiments using HEK293T cells, expressing myc-tagged FAN1 and LB cells expressing endogenous FAN1. As expected, we observed interactions between FAN1 and its known FA-complex interactors, FANCD2 and FANCI (Figure S1A; Table S1; MacKay et al., 2010; Smogorzewska et al., 2010). Interestingly, analysis of the aggregated crosslinking data from both experiments showed multiple proximity areas between FAN1, MLH1, and PMS2, but not MLH3 (Figure 1E; Table S1). Three crosslinks were observed between FAN1 and MLH1, two in the N-terminal part of FAN1 and one in the TPR (tetratricopeptide repeat) domain. We found six crosslinks between FAN1 and PMS2, including four in the N-terminal region of FAN1, one in the TPR domain, and one adjacent to the TPR domain (Figure 1E). Intriguingly, a cluster of four crosslinks between N-terminal FAN1 (p.120-168) and both PMS2 and MLH1 was observed. One of the FAN1 intra-protein crosslinks (K539-S646) was in the structured region of the protein (4RID) at a distance of 27 Å, which is consistent with the maximal distance for the crosslinker used, while all other crosslinks involve unstructured regions with no atomic coordinates present in the Protein Data Bank (PDB). Together, these data show that MutLα, but not MSH3, directly interacts with FAN1 and point to specific contact areas that could be critical for this interaction.

### The FAN1 N-terminal region (p.73-349) mediates its interaction with MLH1 and its effect on CAG stabilization activity

To pinpoint the MLH1-binding region(s) of FAN1, we expressed a series of GFP-tagged FAN1 deletion constructs (Figure 1F) in a well-characterized U2OS cell model stably expressing mutant *HTT* (*mHTT*) exon 1 (Goold et al., 2019). GFP pull-down fractions from cell extracts expressing a FAN1 construct comprising the first 349 residues (FAN1^1–349^) contained levels of MLH1 similar to those produced using full-length FAN1 (FAN1^FL^) (Figures 1G and S1B). In contrast, FAN1^Δ73–349^, a deletion construct missing most of this N-terminal region but retaining the nuclear localization signal (NLS; p.11-25), the ubiquitin-binding zinc-finger domain (UBZ), SAP, TPR, and nuclease domains (Zhao et al., 2014), did not form a complex with MLH1 (Figure 1G). The interaction of the N terminus of FAN1 with MLH1 was confirmed by reverse IP using MLH1 antibodies. This showed FAN1^FL^ and FAN1^1–349^ bind MLH1 (Figure 1H). It is also worth noting that PMS2 partitions with MLH1 in IP fractions derived from FAN1 knockout (FAN1^−/−^), FAN1^FL^, and FAN1^1–349^ cells, indicating FAN1 does not influence the MutLα complex interaction.

To exclude the possibility that deleting a large section of the FAN1 sequence creates an inactive form of the protein that is unable to bind MLH1 because it is misfolded or mis-localized, we performed functional analyses. Live-cell imaging using the FAN1 GFP tag showed exclusively nuclear localization (Figure S1C). Mitomycin C (MMC) stimulates the formation of nuclear FAN1 repair foci in a manner mediated by the UBZ domain and requires FAN1 nuclease activity for ICL repair and survival (MacKay et al., 2010; Smogorzewska et al., 2010). In MMC cell viability assays, as expected, FAN1^1–349^ was present exclusively in the nucleus and formed DNA repair foci, though not as efficiently as the full-length protein, as it lacks the DNA-binding SAP domain, and it provided no protection against MMC toxicity (Figures 1I and S1C–S1E). In turn, FAN1^Δ73–349^ formed repair foci and protected against MMC genotoxicity, indicating that this protein was functional in ICL repair and is therefore unlikely to be misfolded (Figures 1I and S1C–S1E). Thus, the MLH1-binding capacity of these constructs likely reflects the protein’s biological activity rather than mis-localization or misfolding. These data also suggest that the UBZ domain and nuclease activity are not required for the FAN1-MLH1 interaction. To confirm this independently, we expressed the p.C44A/C47A and p.D960A FAN1 mutants, deficient in ubiquitin-binding and nuclease activity, respectively, in U2OS cells and assessed their MLH1-binding capacity using GFP-Trap pull-down assays. Both constructs bound to MLH1 (Figure 1G), and cells expressing these constructs also displayed the expected response to MMC treatment with the p.D960A, but not the p.C44A/C47A variant showing reduced viability (Figures 1I and S1C–S1E).

To assess the effect of the FAN1-MLH1 interaction on CAG repeat instability, we measured CAG repeat expansion over 40 days in isogenic U2OS cells expressing each construct. Introducing the nuclease-deficient p.D960A and p.C44A/C47A UBZ mutations into FAN1^FL^ did not affect the stabilization of the CAG repeat (Figure 1J). FAN1^1–349^ was also able to stabilize the CAG repeat, with a similar expansion rate as FAN1^FL^ (Figures 1J, 1K, and 1L), but FAN1^Δ73–349^, the inverse construct lacking most of the N-terminal region, did not slow CAG expansion as effectively (Figures 1J, 1K, and 1M). Importantly, the expansion rate in FAN1^Δ73–349^ cells was not as fast as FAN1^−/−^ cells, suggesting that a FAN1 region outside of residues 73–349 also contributes to CAG repeat stabilization activity.

Taken together, these structure-function analyses show that the FAN1^73–349^ N-terminal region is necessary and sufficient for interaction with MLH1 and protection against CAG expansion, independent of UBZ and nuclease activity.

### The FAN1 ^126^SPYF^129^ domain mediates MLH1 interaction and confers CAG repeat stabilization in conjunction with FAN1 nuclease activity

**Figure 2:**
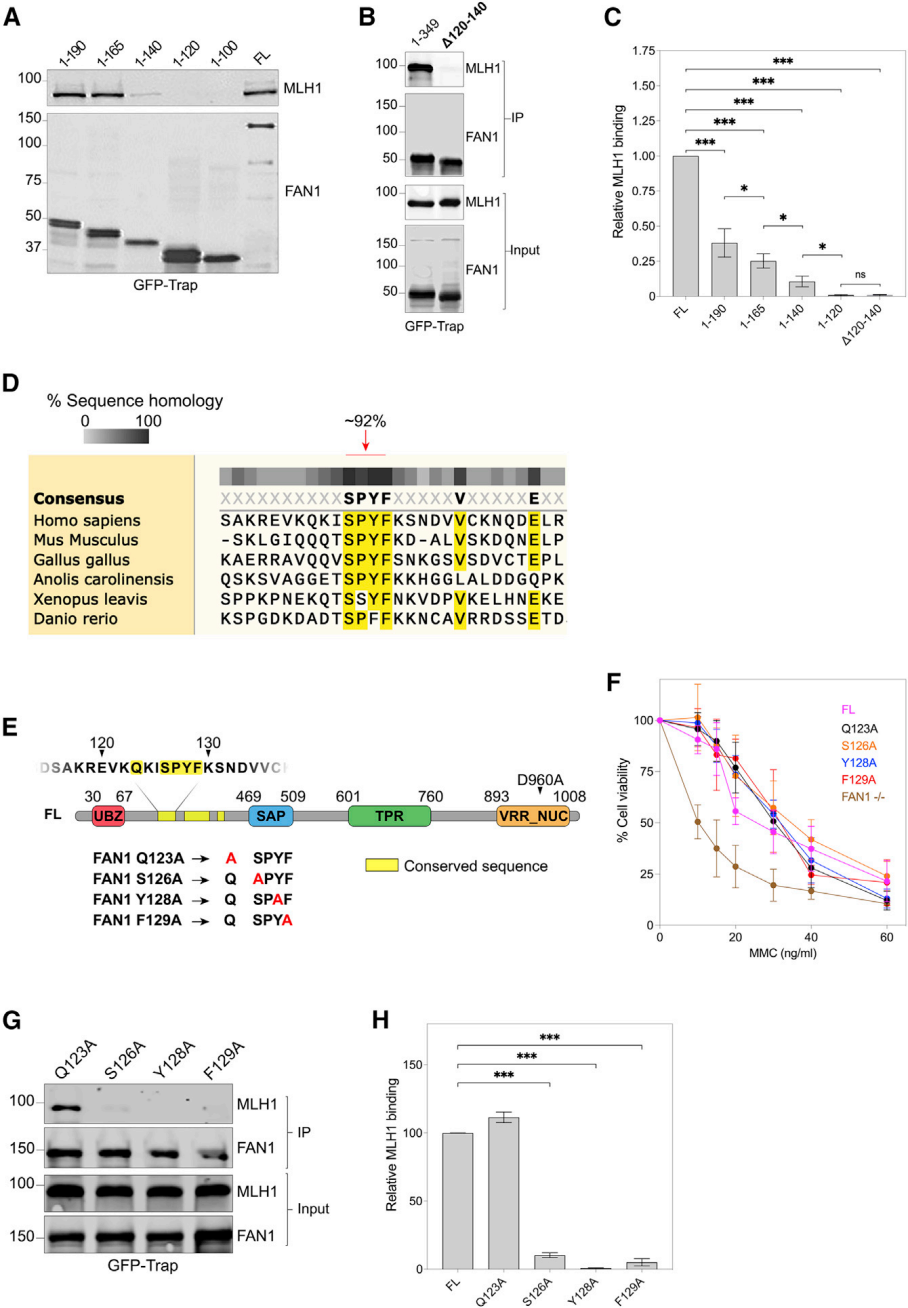
A conserved SPYF motif in FAN1 is required for MLH1 binding (A–C) CoIP extracts using GFP-Trap beads in U2OS cells expressing truncated FAN1 constructs (A and B) with quantification showing progressively longer FAN1 N-terminal fragments bind more MLH1 (C). Note residues 120–140 are essential for MLH1 binding (mean ± SEM, n = 4–5 biological replicates, F(5,22) = 88.31, p < 0.001 by one-way ANOVA with FDR correction of 5%). ^∗^p < 0.05; ^∗∗∗^p < 0.001; ns, non-significant. (D) Conservation analysis schematic showing SPYF motif is heavily conserved within common model species (residues with >80% consensus shown in yellow). (E) Schematic illustrating FAN1 constructs with mutations at conserved SPYF residues that were cloned into the U2OS system. Nuclease null mutation (D960A) is also outlined. UBZ, ubiquitin-binding zinc-finger domain; SAP, SAF-A/B, Acinus and PIAS domain; TPR, tetratricopeptide repeat domain; VRR_NUC, virus-type replication-repair nuclease domain. (F) MMC viability curves in U2OS cells expressing FAN1 SPYF mutants (mean ± SD). Note viability is only reduced in FAN1^−/−^ line (n = 6–8 biological replicates, n = 3 technical replicates) (see also Figure S1G). (G and H) Input and GFP-Trap pull-down fractions from U2OS cell extracts expressing FAN1 SPYF mutants (G) with quantification (H) showing reduced MLH1-binding with mutation of SPYF motif relative to FL construct. Q123A is displayed as a control, having a mutation outside the conserved motif (mean ± SEM, n = 5 biological replicates; F(4,17) = 744.6, p < 0.001 by one-way ANOVA with FDR correction of 5%). ^∗∗∗^p < 0.001.

We observed that FAN1^1–165^ and FAN1^1–190^ constructs both bind MLH1 robustly, but FAN1^1–140^ showed a reduced interaction (Figure 2A). Quantification of GFP-Trap pull-down fractions suggested MLH1 binding increased as the FAN1 N-terminal constructs lengthen, whereas FAN1^1–120^ and the deletion construct FAN1^Δ120–140^ showed little or no MLH1 binding (Figures 2A–2C). Therefore, MLH1 binding absolutely requires FAN1 residues 120–140, but downstream sequences could contribute to complex stability. These data are consistent with on-bead crosslinking experiments that showed close associations between MLH1-PMS2 and the N-terminal region of FAN1 (Figure 1E).

The N-terminal region of FAN1 is largely unstructured and relatively nonconserved. It does, however, contain three highly conserved regions, the first of which consists of a SPYF motif (p.126-129; Figure 2D) similar to the MLH1-interacting peptide box (MIP-box) found in many of MLH1’s interaction partners (Dherin et al., 2009; Iyer et al., 2010). Considering the similarity to a known MLH1-binding sequence and the data from our structure-function analysis, we explored the role of ^126^SPYF^129^ in the FAN1-MLH1 interaction. We introduced a series of alanine substitutions into the SPYF motif using site-directed mutagenesis and expressed these mutants as GFP fusion proteins in U2OS cells (Figure 2E). Importantly, cells expressing these constructs were protected against MMC toxicity and formed nuclear repair foci normally, suggesting the SPYF mutations did not affect ICL repair activity (Figures 2F, S1C, S1F, and S1G). Instead, GFP-Trap pull-down fractions showed residues within the SPYF motif, in particular the aromatic residues Y128 and F129, as critical for MLH1 binding, whereas mutation of a well-conserved residue outside this sequence (Q123) did not affect binding (Figures 2G and 2H). These data agree closely with our structure-function analysis and demonstrate that FAN1 interacts with MLH1 through its conserved N-terminal SPYF motif.

**Figure 3:**
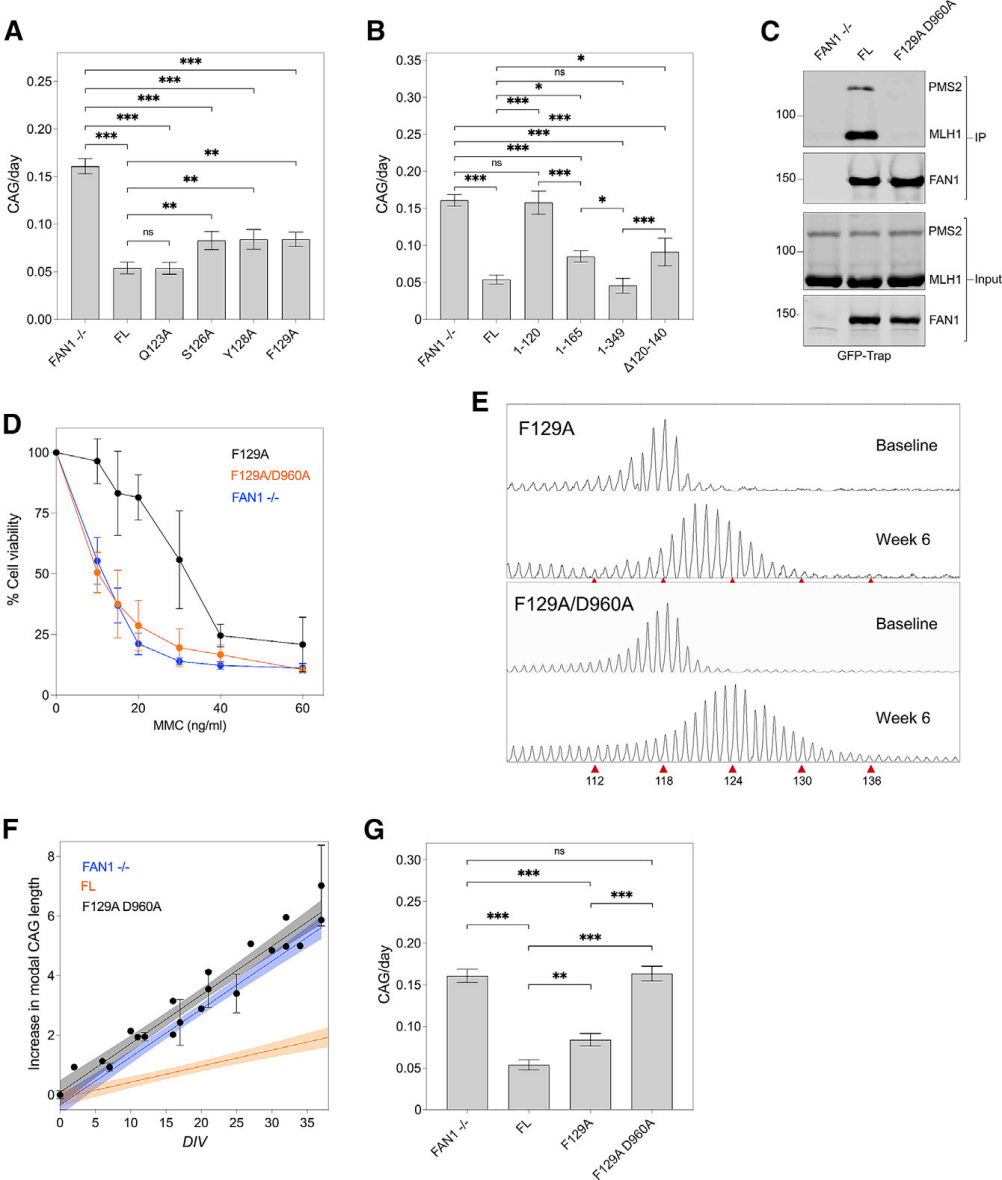
FAN1 SPYF motif and nuclease activity stabilize the *HTT* CAG repeat (A) CAG expansion rates in U2OS cells expressing FAN1 constructs with mutations at conserved SPYF motif. Note that mutation of this domain results in hastened expansion of the *HTT* CAG repeat. Q123A is displayed as a control, having a mutation outside the conserved motif. (mean ± SEM, n = 2–5 biological replicates, n = 3–6 technical replicates, F(5,83) = 28.64, p < 0.001 by one-way ANOVA with FDR correction of 5%). ^∗∗^p < 0.01, ^∗∗∗^p < 0.001, ns = non-significant. (B) CAG expansion rates in U2OS cells expressing truncated N-terminal constructs of FAN1, showing residues 120–140 contribute significantly to *HTT* CAG repeat stability. (mean ± SEM, n = 2–5 biological replicates, n = 3–6 technical replicates, F(5,86) = 22.38, p < 0.001 by one-way ANOVA with FDR correction of 5%). ^∗^p < 0.05, ^∗∗∗^p < 0.001, ns = non-significant. (C) Input and GFP-Trap pull-down fractions from U2OS cell extracts expressing FAN1^FL^ and FAN1^F129A/D960A^ showing reduced MLH1-binding with mutation of SPYF motif relative to FL. Note equivalent FAN1^FL^ and FAN1^F129A/D960A^ expression (n = 2 biological replicates). (D) MMC viability curves in U2OS cells expressing FAN1^F129A^ and FAN1^F129A/D960A^ mutants (mean ± SD, n = 6–7 biological replicates, n = 3 technical replicates). Note resistance to MMC toxicity is only maintained in the F129A line. See also Figure S1H. (E–G) Fragment analysis traces illustrating expansion of the exogenous *HTT* 118 CAG repeat in U2OS cells expressing FAN1^F129A^ or FAN1^F129A/D960A^ mutants over 6 weeks in culture with time courses plotted (F; mean ± SD, 95% CI in shaded areas) and quantified (G). Cells expressing FAN1^F129A/D960A^ show equivalent expansion as FAN1^−/−^ cells (mean ± SEM, n = 2–5 biological replicates, n = 3–6 technical replicates, F(3,72) = 39.27, p < 0.001 by one-way ANOVA with FDR correction of 5%). ^∗∗^p < 0.01; ^∗∗∗^p < 0.001; ns, non-significant.

Mutations S126A, Y128A, and F129A within the SPYF motif reduced the stabilization activity of FAN1, with substitution of the aromatic residues exhibiting the greatest increase in the CAG repeat expansion rate, while Q123A had no effect (Figure 3A). Similarly, FAN1^1–120^ did not stabilize the CAG repeat, while longer SPYF-motif-containing constructs, including FAN1^1–165^, significantly restrained CAG expansion (Figure 3B). Consistent with this, deleting residues 120–140 (FAN1^Δ120–140^) from the FAN1^1–349^ construct reduced the stabilization activity (Figure 3B). As for the SPYF mutants, CAG repeat stabilization activity and MLH1-binding correlate closely, indicating they are mechanistically linked.

Mutation of the SPYF motif was associated with an increased expansion rate relative to FAN1^FL^, significant because it shows nuclease function alone does not fully stabilize the CAG repeat, as FAN1^FL^ and SPYF mutants have similar ICL repair activity (Figures 2F, S1C, S1F, and S1G). Despite this, we observed that the expansion rate in SPYF-deficient constructs was not as fast as in FAN1^−/−^ cells (Figure 3A). In fact, the stabilization activity of the SPYF mutants was similar to that shown by FAN1^Δ73–349^, suggesting there is residual stabilization activity downstream of p.349, with the most likely candidate being the nuclease domain. To assess this, we introduced the nuclease-deficient p.D960A mutation into a SPYF-deficient construct (FAN1^F129A^). Immunoblots demonstrated that FAN1^F129A/D960A^ and FAN1^FL^ were expressed at similar levels (Figure 3C), and FAN1^F129A/D960A^ was able to form DNA repair foci in response to MMC, a response requiring a functional UBZ domain (Figures S1C and S1F). However, as expected, GFP-Trap pull-down experiments demonstrated reduced MLH1 binding, while decreased MMC viability showed deficient ICL repair (Figures 3C, 3D, and S1H). Importantly, repeat expansion in FAN1^F129A/D960A^ cells was faster than the F129A single mutant and equivalent to FAN1^−/−^ cells (Figures 3E–3G).

Taken together, these data show that the FAN1 SPYF motif mediates its MLH1 interaction and that FAN1’s protective stabilization of the CAG repeat involves MLH1 binding and the nuclease domain.

### FAN1 regulates MMR activity by competing with MSH3 for MLH1 binding

**Figure 4:**
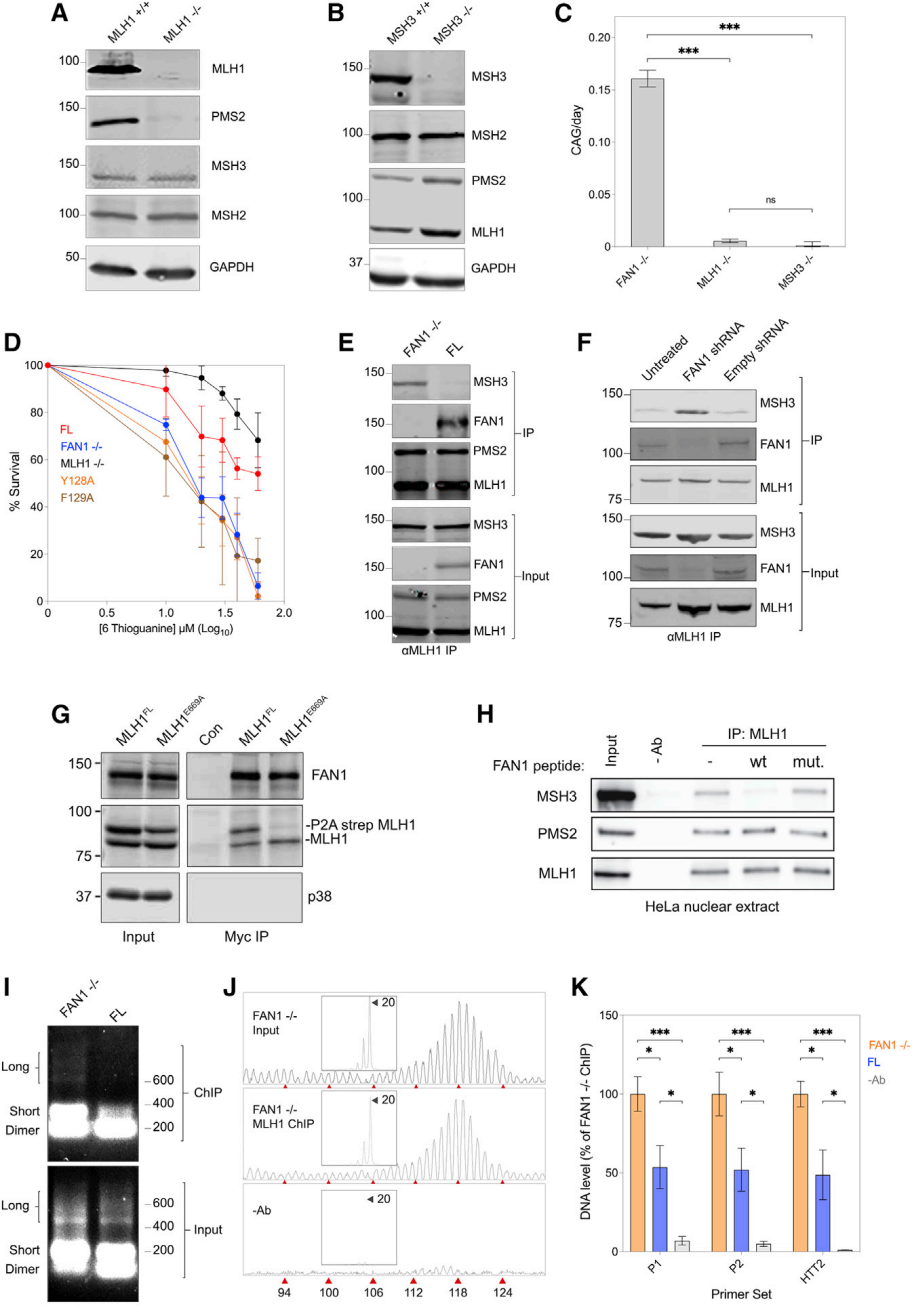
FAN1 regulates mismatch repair (MMR) activity through MLH1 binding (A and B) Western blots showing MMR protein expression in U2OS *MLH1* (A) and *MSH3* (B) knockout lines (n = 3 biological replicates). (C) CAG expansion rates in FAN1^−/−^, MLH1^−/−^, and MSH3^−/−^ U2OS cell lines. Note that knockout of *MSH3* or *MLH1* ablates CAG repeat expansion (mean ± SEM, n = 2–5 biological replicates, n = 3–6 technical replicates, F(2,72) = 272.5, p < 0.001 by one-way ANOVA with FDR correction of 5%). ^∗∗∗^p < 0.001; ns, non-significant. (D) 6TG viability curves in U2OS cells expressing FAN1 constructs and MLH1, showing cells with an intact FAN1 SPYF motif have enhanced resistance to 6TG, indicating reduced MMR activity. MLH1^−/−^ cells serve as a control (mean ± SD, n = 5 biological replicates, n = 3 technical replicates). See also Figure S2A. (E) CoIP of MLH1 and binding partners from FAN1^−/−^ and FAN1^FL^ cells. Note FAN1 expression reduces MSH3 levels in MLH1 IP fractions but does not affect PMS2 (n = 4 biological replicates). See also Figure S2F. (F) CoIP of MLH1 and binding partners from 125 CAG HD MSNs with shRNA-mediated FAN1 knockdown. Untreated cells and an empty shRNA vector were used as controls. Note that FAN1 knockdown increases MSH3 levels in MLH1 IP fractions (n = 3 biological replicates). See also Figures S2G and S2H. (G) CoIP of myc-tagged FAN1 from HEK293T cells expressing strep-tagged MLH1 variants and endogenous MLH1. Note endogenous MLH1 and strep-tagged MLH1^FL^ bind to FAN1, whereas MLH1^E669A^ does not (n = 2 biological replicates). (H) FAN1 peptide competition assay in HeLa cell nuclear extracts showing FAN1 wild-type (wt) 60-mer peptides (15 μM) reducing MLH1-MSH3 interactions, whereas FAN1 mutant (mut.) peptides do not (n = 3 biological replicates). (I) ChIP extracts from FAN1^FL^ and FAN1^−/−^ U2OS cells, immunoprecipitated with αMLH1 antibodies and DNA amplified with primers targeting *HTT* CAG repeat region. Note the decreased levels of both long (exogenous *HTT*) and short (endogenous *HTT*) amplicons in FL ChIP fractions (n = 3 biological replicates). (J) Fragment analysis traces from U2OS FAN1^−/−^ extracts show the presence of the CAG repeat from the endogenous *HTT* allele (20 CAG units) and the longer exogenous repeat (118 CAG) from the exon 1 construct in both input and ChIP fractions. The lack of signal in the control IP (-Ab) shows the specificity of the procedure (n = 3 biological replicates, n = 3 technical replicates). (K) Quantification of DNA levels in ChIP fractions from FL and FAN1^−/−^ U2OS cells. Primer pairs proximal to the CAG repeat (P1 and P2) and toward the 3′ end of *HTT* (HTT2) were used (mean ± SEM, n = 3 biological replicates, n = 3 technical replicates; P1: F(2,6) = 20.76, p = 0.002; P2: F(2,6) = 17.84, p = 0.003; HTT2: F(2,6) = 23.56, p = 0.001 by one-way ANOVA with FDR correction of 5%). ^∗^p < 0.05; ^∗∗∗^p < 0.001.

Consistent with reduced MMR activity, *MLH1* and *MSH3* knockout abolishes repeat expansion (Figures 4A–4C) and in the case of *MLH1* increases resistance to 6-thioguanine (6TG) (Figures 4D and S2A; Swann et al., 1996). Surprisingly, we observed that expression of FAN1^FL^ or FAN1^1–349^ in an MLH1^WT^ background also increases 6TG resistance relative to FAN1^−/−^ cells, whereas expression of FAN1^Δ73–349^ or SPYF mutants had no effect (Figures 4D and S2A–S2C). This suggested that the SPYF motif sequesters MLH1 away from its other binding partners, reducing MMR activity and ultimately preventing repeat expansion. To explore this possibility, we tested the ability of MLH1 to associate with MSH3 in the presence or absence of FAN1. This is of particular significance, given the key role of MSH3 in somatic expansion (Figures 4B and 4C) and the similarity of the FAN1 SPYF motif to the MIP-box in MSH3, which mediates binding to MLH1. Consistent with this, MSH3 pull-downs showed that MLH1 levels were reduced in FAN1^FL^ samples relative to FAN1^−/−^ (Figures S2D and S2E). FAN1 was not observed in these IPs, confirming it does not interact directly with MSH3. This suggests that FAN1 controls MMR complex assembly by sequestering MLH1. In MLH1 pull-downs, FAN1, MSH3, and PMS2 were recovered. The presence of FAN1 did not affect PMS2 levels, suggesting it does not interfere with MutLα complexing, but MSH3 levels were reduced in FAN1^FL^ relative to FAN1^−/−^ samples (Figures 4E and S2F). Thus, FAN1 expression reduces the MLH1-MSH3 interaction. To show this relationship exists in cells expressing endogenous proteins, we knocked down FAN1 expression in HD iPSCs carrying 125 CAGs. Stable incorporation of small hairpin RNA (shRNA) targeting *FAN1* reduced FAN1 protein levels by 90%–95% (Figure S2G), and medium spiny neurons (MSNs) derived from these cells show increased CAG repeat expansion rate relative to control cells (data not shown). MLH1 IPs from MSN extracts show FAN1 knockdown consistently increased the levels of MSH3 in the IP fractions relative to the control cells (Figures 4F and S2H). Thus, we conclude that like in U2OS cells, FAN1 expression reduces the MLH1-MSH3 interaction in HD MSNs.

The MLH1 MIP-box-interacting (S2) site contains several key residues spread across the C-terminal domain. Mutation of one of these, E669, in the human sequence has been shown to disrupt MLH1 MIP-box interactions but leave MutL complex formation unaffected (Dherin et al., 2009; Iyer et al., 2010). In the U2OS system, myc-tagged FAN1^FL^ binds strep-tagged MLH1 with wild-type (WT) sequence, but not the E669A mutant (Figure 4G), indicating the SPYF motif acts as a canonical MIP-box. This supports our finding that FAN1 competes with MSH3 for the MLH1 S2 interaction site. Incubating HeLa nuclear extracts with a synthetic 60-mer FAN1 WT peptide surrounding the SPYF motif reduced the levels of MSH3 co-immunoprecipitating with MLH1 in a dose-dependent manner (Figures 4H and S2I). Critically, peptides in which the MLH1 interaction site is mutated did not affect the MLH1-MSH3 interaction (Figure 4H).

Taken together, our data support a model where FAN1 competes with MSH3 for binding the MIP-box-interacting S2 domain of MLH1. In human striatum and cortex, MLH1 and FAN1 are expressed at similar levels, both higher than the levels of MSH3 (Figure S3A), indicating FAN1 could be a major regulator of MLH1-MSH3 interactions *in vivo*.

One consequence of this may be a reduction of MSH3-dependent MLH1 recruitment to the CAG repeat. To assess this, we performed a chromatin IP (ChIP) assay, involving anti-MLH1 IP from FAN1^−/−^ and FAN1^FL^ U2OS cells. PCR across the *HTT* CAG repeat identified the endogenous 20 CAG (“short”) and exogenous 118 CAG repeat (“long”) in both samples (Figure 4I). The presence of long and short repeat sequences was confirmed by fragment analysis of the ChIP samples (Figure 4J). qRT-PCR analysis showed there was less *HTT* CAG DNA in anti-MLH1 ChIP fractions from FAN1^FL^ cells relative to FAN1^−/−^ (Figure 4K). This is consistent with FAN1 reducing MLH1’s interaction with the CAG repeat.

To further explore the role of DNA repair genes implicated in somatic instability, we analyzed FAN1^−/−^, FAN1^FL^, MLH1^−/−^, and MSH3^−/−^ U2OS cell lines for evidence of microsatellite instability (MSI) over the course of our CAG repeat expansion assays.

MutSβ deficiency results in MSI at tetra- and dinucleotide repeats, whereas MutSα deficiency causes MSI at mono- and dinucleotide repeats (Carethers, 2017). Although MLH1^−/−^ cells did not demonstrate CAG repeat expansion (Figure 4C), there was instability at tetranucleotide marker D20S85 (otherwise known as EMAST, or elevated microsatellite alterations at selected tetranucleotide repeats), indicating MMR deficiency (Figure S3B). Similarly, MSH3^−/−^ cells showed MSI at several tetranucleotide (MYCL1, D9S242, D20S82, and D20S85) and dinucleotide loci (D8S321), indicating MutSβ deficiency, but the CAG repeat remained stable (Figures 4B, 4C, and S3B). Manipulation of FAN1 did not affect MSI in the time course of the assay (Figure S3B). Collectively, these data suggest that FAN1 suppresses MMR activity by sequestering MLH1 away from MSH3, thus preventing error-prone repair and CAG repeat expansion.

## Discussion

Recent genetic studies have shown somatic expansion of the CAG repeat is the key pathogenic process driving HD onset and progression. In this study, we investigated the interaction of the HD genetic modifiers FAN1 and MLH1 and their role in repeat instability in patient-derived cells, HD mouse models, and a U2OS cell system stably expressing *mHTT* exon 1. We demonstrated that a FAN1 SPYF motif (p.126-129) mediates its binding to MLH1 and that this interaction protects against CAG repeat expansion. We also demonstrated the nuclease domain of FAN1 is involved in the protective effects of FAN1.

FAN1 N-terminal deletion constructs lacking the SPYF motif fail to stabilize the CAG repeat; FAN1^1–120^ accelerates repeat expansion to the same rate as FAN1^−/−^, whereas longer constructs containing the SPYF motif, including FAN1^1–165^, slow the expansion rate significantly. Consistent with this, deleting residues 120–140 (FAN1^Δ120–140^) from the FAN1^1–349^ construct reduces stabilization activity. SPYF mutations reduce FAN1-MLH1 binding and accelerate repeat expansion. MLH1 binding, and CAG stabilization activity correlate closely, indicating they are mechanistically linked (Figures 2 and 3). The homology between the FAN1 SPYF and MSH3 MIP-box supports our hypothesis of competition for MLH1 binding. A MIP-box is found in several MLH1 interaction partners, including MSH3, EXO1, and NTG2, and it has been shown to interact with the C-terminal S2 site of MLH1, a region comprising several conserved residues (Dherin et al., 2009; Iyer et al., 2010). Our crosslinking results show that interactions between the FAN1 SPYF motif and MLH1 are clustered at the unstructured central domain of MLH1 and include crosslinks consistent with an interaction near the S2 site. Introducing the E669A mutation into the S2 site of MLH1 abrogates FAN1 interaction, suggesting the SPYF motif indeed acts as a MIP-box. FAN1 binding would therefore sterically inhibit MLH1’s interaction with MSH3 and modulate MutSβ-driven MMR activity. The close associations among FAN1, MLH1, and PMS2 demonstrate that FAN1 interacts functionally with the MutLα complex. CoIP shows MLH3 also associates with FAN1 in U2OS cell extracts, suggesting the MutLγ complex may also interact with FAN1.

Consistent with previous data from mouse models, we find that MLH1 or MSH3 knockout prevents CAG repeat expansion (Figures 4A–4C), showing the absolute requirement of MutSβ-driven MMR for this process (Loupe et al., 2020; Pinto et al., 2013; Tomé et al., 2013). Our data suggest that FAN1 competes with MSH3 for MutLα (or MutLγ) binding, preventing MMR-driven CAG expansion. The potential significance to HD is shown by the inhibitory effect of FAN1 expression on MLH1-MSH3 interactions in HD MSNs and the similar expression levels of MLH1 and FAN1 in human cortex and striatum, meaning FAN1 could be a major regulator of MLH1-MSH3 interactions *in vivo*.

Cells defective in MMR are resistant to 6TG toxicity and display MSI (Swann et al., 1996). MLH1^−/−^ U2OS cells are resistant to 6TG and show instability at an EMAST locus in the genome (Figures 4D, S2A, and S3B), indicating they have dysregulated MMR activity. Interestingly, cells overexpressing FAN1 with an active SPYF domain showed significantly increased resistance to 6TG, as compared to FAN1^−/−^ cells (Figures 4D and S2A–S2C). These cells did not show alterations at EMAST loci, which likely reflects the partial inhibition of MMR activity and the relatively short time course of the assay. Importantly, FAN1 constructs lacking an active SPYF motif did not protect against 6TG toxicity, showing that MLH1-binding likely underlies FAN1’s regulation of MMR activity. This is interesting as it suggests FAN1 may be modulating both MutSα- and MutSβ-driven MMR activity (Stojic et al., 2004). Our data show that FAN1 sequesters MLH1 and prevents interaction with MSH3 by competing for the MIP-box-binding S2 site (Figures 4G and 4H). The lack of MSH2 and MSH6 in anti-FAN1 IP fractions confirms earlier reports that these proteins do not directly interact (MacKay et al., 2010; Smogorzewska et al., 2010) and suggests a similar mechanism may operate to regulate MutSα-MLH1 interactions. MMR interactions with the FA pathway and FAN1 itself have been reported previously (Peng et al., 2014; Williams et al., 2011; Rikitake et al., 2020), but direct inhibition of MMR, mediated by MLH1 sequestration, has not. The physiological significance of these findings needs further inquiry, but it is evident from experiments in mouse models that FAN1 and MLH1 interact genetically and play a crucial role in regulating somatic expansion, likely by modulating MMR activity (Loupe et al., 2020).

Our data also indicate that the FAN1 nuclease domain contributes to its repeat stabilization activity, in accordance with recent data in preprint on *bioRxiv* (McAllister et al., 2021). The FAN1^F129A/ D960A^ SPYF and nuclease double mutant demonstrated that FAN1-MLH1 binding and nuclease activity have independent, but additive, repeat-stabilizing effects. Though the p.D960A nuclease inactivation alone did not affect repeat instability (Goold et al., 2019; Figure 1J), the overexpression of FAN1 mutants in U2OS cells likely masked the subtle contribution of the nuclease domain by sequestering most available MLH1 and shutting down error-prone MMR. In the absence of this dominant activity, for example following SPYF mutation, the stabilization activity of the nuclease domain can be observed. In this scenario, FAN1’s nuclease activity could operate downstream of MSH3-mediated recruitment of MLH1, regulating the repair process to reduce errant CAG incorporation, possibly by acting directly on the DNA. This proposal is supported by data showing FAN1 binds directly to CAG repeat DNA (Goold et al., 2019; Kim et al., 2020). Our data also suggest an intact FAN1 UBZ domain is not required to stabilize the CAG repeat. Mutations that inactivate ubiquitin binding do not affect the expansion rate but prevent recruitment to DNA repair foci induced by MMC treatment. This indicates recruitment by the ID2 FA complex is not required for stabilization.

### Limitations of the study and future directions

Further studies are needed to establish the processes modulating the FAN1-MLH1 interaction. For instance, our FAN1 structure-function analyses were performed using cells overexpressing exogenous FAN1. It will be important to confirm these interactions in the context of endogenous FAN1 in human cells and by creating FAN1 mouse models. Moreover, the precise requirement for the FAN1 nuclease activity in this model will need to be (re)assessed in more detail. It is compelling to speculate that, as for EXO1 (Guan et al., 2021), MLH1 could directly regulate FAN1 nuclease activity to promote DNA resection. A greater understanding of such processes will also be critical for the development of new therapies.

## STAR★Methods

### Key resources table

**Table:** 

REAGENT or RESOURCE	SOURCE	IDENTIFIER
**Antibodies**		

FAN1 sheep polyclonal (human)	CHDI Foundation	N/A
FAN1 sheep polyclonal (mouse)	MRC-PPU reagents	S101D
MLH1 mouse monoclonal (human)	BD Biosciences	554073: RRID:AB_395227
MSH3 mouse monoclonal	BD Biosciences	611390; RRID:AB_398912
MSH2 rabbit	Cell Signaling Technology	2017;RRID:AB_2235387
MSH6 mouse monoclonal	BD Biosciences	610918;RRID:AB_398233
MLH1 mouse monoclonal (human/mouse)	Abcam	ab92312;RRID:AB_2049968
PMS2	Santa Cruz Biotechnology	sc-25315; RRID:AB_628163
MLH3	Santa Cruz Biotechnology	sc-25313; RRID:AB_627954
PCNA	Cell Signaling Technology	13110; RRID:AB_2636979
GAPDH	Santa Cruz Biotechnology	sc-32233; RRID:AB_627679
GFP	Santa Cruz Biotechnology	sc-9996; RRID:AB_627695

**Bacterial and virus strains**		

One Shot TOP10 *E. coli*	ThermoFisher	C4040

**Chemicals, peptides, and recombinant proteins**		

FAN1 60-mer wild-type MLH1-interaction-defective mutant peptides (amino acids 118-177)	GenScript	N/A

**Critical commercial assays**		

Thiazolyl Blue Tetrazolium Bromide (MTT) assay	Sigma	34-000-1002
SYBR Green Master Mix	ThermoFisher	A25741
QuickChange XL kit	Agilent	200516
QIAamp DNA Mini kit	QIAGEN	51306

**Deposited data**		

Mass spectrometry proteomics data	ProteomeXchange	PXD023221

**Experimental models: Cell lines**		

U2OS FAN1^−/−^ cells	Prof Rouse University of Dundee)	N/A
iPSC 125 CAG	This study	N/A
HEK293T	ATCC	N/A
Lymphoblastoid cells	This study	N/A
PheonixiAmpho	ATCC	N/A

**Experimental models: Organisms/strains**		

zQ175 mice	Charles River	N/A
R6/2 mice	Envigo, Netherlands	N/A

**Oligonucleotides**		

qRT-PCR primers pair 1 forward CCGCTCAGGTTCTGCTTTTA,	Thermo	N/A
pair 1 reverse GCCTTCATCAGCTTTTCCAG	Thermo	N/A
pair 2 forward CCAGAGCCCCATTCATTG	Thermo	N/A
pair 2 reverse GCCTTCATCAGCTTTTCCAG	Thermo	N/A
3′ *HTT* forward TGCCTTTCGAAGTTGATGCA,	Thermo	N/A
3′ *HTT* reverse TGCCACCACGAATTTCACAA).	Thermo	N/A
Fragment analysis	Thermo	N/A
6-FAM-labeled forward primer: AAGGCCTTCGAGTCCCTCAAGTCCTT.	IDT	N/A
Reverse primer: CGGCTGAGGCAGCAGCGGCTGT	IDT	N/A

**Recombinant DNA**		

pcDNA5.1 FRT/TO GFP FAN1	MRC-PPU reagents	DU19495
pcDNA5.1 FRT/TO GFP FAN1 deletion constructs and mutants	This study	N/A

**Software and algorithms**		

GraphPad Prism 9	Software, Inc, USA	https://www.graphpad.com/scientific-software/prism/
GeneMapper v6. software	ThermoFisher	A38888
Fragment analysis Custom R script	This study	https://caginstability.ml
xQuest/xProphet	xQuest	N/A

**Other**		

GFP-Trap beads	ChromoTek	gtma-10
Protein G magnetic beads (Dynabeads)	Thermo-Fisher Scientific	10003D

### Resource availability

#### Lead contact

Further information and requests for resources and reagents should be directed to and will be fulfilled by the lead contact, Prof Sarah J. Tabrizi (s.tabrizi@ucl.ac.uk).

#### Materials availability

iPSC 125 CAG were generated from peripheral blood mononuclear cells donated by a HD patient by reprogramming at Censo in Edinburgh, UK. iPSCs were karyotypically stable, and whole genome sequencing of blood from the same individual and iPSCs did not identify any clinically significant variants.

### Experimental model and subject details

#### Mice

All procedures were performed in accordance with the Animals (Scientific Procedures) Act 1986 and were approved by the University College London Ethical Review Process Committee. R6/2 mice were bred by backcrossing R6/2 males to C57BL/6JOlaHsd x CBA/CaOlaHsd F1 females (B6CBAF1/OlaHsd, Envigo, Netherlands) and zQ175 mice were bred by backcrossing males to C57BL/6J females (Charles River). Mouse husbandry, health status, genotyping and CAG repeat sizing were as previously described (Landles et al., 2020). For IP experiments, brains were from female mice. CAG size for the zQ175 was 206 (SD ± 3) and R6/2 was 182 (SD ± 1). Mice were sacrificed by a schedule 1 procedure at 6 months of age (zQ175) or 12 weeks of age (R6/2), brains were dissected rapidly, tissues were snap frozen in liquid nitrogen and stored at −80°C.

### Method details

#### Cell culture and manipulation

U2OS FAN1^−/−^ cells were generated as previously described, featuring FRT sites introduced into the genome, enabling complementation with tetracycline-inducible FAN1 variants when co-transfected with Flp recombinase. This line was kindly gifted by Prof. John Rouse (University of Dundee, Scotland). Introducing a lentiviral *HTT* exon 1 construct harboring 118 CAG repeats allows examination of the effects of different FAN1 activities/regions on repeat stability (Goold et al., 2019). U2OS cells were maintained in DMEM with GlutaMAX, supplemented with 10% FBS and pen-strep. ICL repair assays were performed as described previously (Goold et al., 2019). Cells were plated at 200 cells per well in a 96 well plate. The next day MMC was added to cells at increasing concentrations for 16 h. Cells were washed into fresh media, cultured for 7-10 days until control cells were confluent. The proportion of live cells was then assayed. Cell survival was expressed as a percentage of control untreated cells. For quantifying GFP-FAN1 foci, cells were imaged using a fluorescent microscope and were considered positive with ≥ 5 foci per nucleus.

Lymphoblastoid cells derived from the TRACK-HD cohort were cultured in RPMI medium supplemented with 15% fetal bovine serum (FBS), 100 U/ml penicillin and 100 μg/ml streptomycin.

The shRNA hairpin targeting FAN1 (target sequence: GTAAGGCTCTTTCAACGTA) was subcloned into pSUPER.retro.Puro and transfected into Phoenix Ampho packaging cells using Lipofectamine LTX. After 16 h, 8 mL fresh media was added. Cell media containing mature retrovirus was harvested 48 h post-transfection. This was filtered and frozen at −80°C or used directly.

#### iPSC culture and manipulation

Stem cells were maintained in Essential E8 medium (ThermoFisher) on Thermo-Nunc plasticware coated with Geltrex (GIBCO) diluted 1:50 in DMEM/F12 without glutamine. They were passaged by manual dissociation using 0.02% EDTA (GIBCO). MSN differentiation was carried out as described (32) using Activin A to direct ganglionic/striatal fate. Media containing retrovirus encoding shRNA hairpins targeting FAN1 or empty vector was mixed one to one with normal iPSC media and supplemented with polybrene (8 μg/ml). This media was added to iPSC at ∼70% confluence and the cells were incubated for 16 h. Fresh media was added to the cells for a further 48 h prior to selection. For this, the media was supplemented with puromycin (1 μg/ml) and the cells were monitored ensuring regular media changes to minimize the number of dead cells in the culture. Colonies of transduced cells were detected after 2–3 weeks. Untreated cells were cultured alongside the selected cells and used as controls in subsequent experiments.

#### Immunoprecipitation, ChIP, cloning, SDM and CRISPR

ChIP analysis was performed with the EZ-Magna ChIP A Chromatin Immunoprecipitation Kit according to the manufacturer’s instructions. Chromatin was fragmented by 15 cycles of 30 s sonication in a Bioruptor apparatus at 4°C. Immunoprecipitation was done overnight at 4°C using anti-MLH1 antibodies (BD Biosciences). DNA from ChIP and input fractions was quantified by SYBR (Thermo, #A25741) qRT-PCR using primers targeting two regions proximal to the CAG repeat (pair 1 forward CCGCTCAGGTTCTGCTTTTA, reverse GCCTTCATCAGCTTTTCCAG; pair 2 forward CCAGAGCCCCATTCATTG, reverse GCCTTCATCAGCTTTTCCAG), and one distal, at the 3′ end of *HTT* (forward TGCCTTTCGAAGTTGATGCA, reverse TGCCACCACGAATTTCACAA). DNA levels were quantified relative to a genomic DNA standard. Results were expressed as percentage of the DNA levels in U2OS FAN1^−/−^ ChIP fractions.

Cell extracts were prepared for SDS-polyacrylamide gel electrophoresis (PAGE) as described previously (Goold et al., 2011). Cells were detached by trypsinisation, washed in media and centrifuged at 300 g for 5 min. Cell pellets were resuspended in PBS, transferred to 1.5 mL eppendorfs and centrifuged at 10,000 g for 1 min. Cell pellets were resuspended in IP buffer (20 mm Tris, pH 7.4, 150 mm NaCl, 1 mm EDTA, 1% Triton X-100 supplemented with Benzonase 2 U/ml and protease inhibitors) and incubated on ice for 20 min. Protein concentrations in the lysates were determined by Bio-Rad assay. Proteins were precipitated with cold methanol and resuspended in SDS sample buffer to 2 mg/ml. The antibodies used were a FAN1 sheep polyclonal antibody (Goold et al., 2019); MSH3 or MLH1 monoclonal antibodies (BD Biosciences, UK); PCNA and MSH2 (Cell Signaling Technology, Danvers, MA, USA); and PMS2, GAPDH and GFP rabbit polyclonal antibodies (Santa Cruz Biotechnology, Dallas, TX, USA). To analyze mouse cortex samples antibodies to FAN1 (sheep polyclonal S101D, available from Dundee MRC PP) and MLH1 (Abcam) were used. Immunoblots were quantified with the Odyssey CLx Imaging System, (Lincoln, NE, USA) using Glyceraldehyde 3-phosphate dehydrogenase (GAPDH), p38 MAP kinase and β-actin as loading controls. For immunoprecipitation (IP) analysis, washed cells were resuspended in IP buffer and incubated on ice for 20 min. The cell extracts were centrifuged at 10,000 g for 2 min and the supernatant fraction was used as input. Mouse cortex was homogenized in modified RIPA buffer (50mM Tris pH 7.4, 1mM EDTA, 1% Tx100, 0.5% sodium deoxycholate and 0.1% SDS supplemented with Benzonase 2 U/ml and protease inhibitors) and incubated on ice for 20 min. The sample was clarified by centrifugation at 20,000 g for 10 min at 4°C. Extracts were diluted 1 to 10 with IP buffer and used as input. GFP-Trap beads or FAN1 sheep polyclonal and MSH3 or MLH1 monoclonal antibodies and protein G magnetic beads were used to capture protein complexes. Beads were washed 3 times in IP buffer and eluted by heating in SDS sample buffer.

#### Peptide competition assay

Custom-designed FAN1 60-mer wild-type and MLH1-interaction-defective mutant peptides (amino acids 118-177) were purchased from GenScript (A.P., unpublished data). 1 mg of HeLa nuclear extracts were incubated with or without peptides in 0.5 mL NP40 buffer for 2 h at 4°C with rotation. 1 μg of anti-MLH1 rabbit monoclonal antibody (D38G9, Cell Signaling) was added to the samples and incubated overnight at 4°C with rotation. Protein A-Sepharose beads (CL4B Sigma) were equilibrated in NP40 buffer and 25 μl bead slurry were then added to each sample and incubated for 2 h at 4°C with rotation. The beads were then washed three times with NP40 buffer and once with 1xTEN100 buffer, boiled in SDS sample buffer and analyzed by western blotting using anti-MSH3 (H300, sc-11441, Santa Cruz), anti-PMS2 (B3, sc-25315, Santa Cruz) and anti-MLH1 (ab92312, Abcam) antibodies.

FAN1 point mutations were generated by site-directed mutagenesis using the QuickChange XL kit according to the manufacturer’s instructions (Agilent, CA, USA). The presence of the DNA base changes was confirmed by sequencing of the genomic DNA isolated from reconstituted cells. Deletion constructs were synthesized by GeneArt (Thermo Fisher) and subcloned into pcDNA5.1 FRT/TO GFP FAN1 using BamH1, EcoRV and Not1 restriction sites. A biscistronic vector encoding myc-tagged FAN1 downstream of a strep tagged MLH1 and separated by a P2A sequence was generated. The E669A substitution was generated by SDM. CRISPR guide sequences encoded in pX458 vector were used to inactivate the MSH3 and MLH1 genes in U2OS cells. Knockout was confirmed by western blot, sequencing and functional assays.

#### Somatic instability assay

DNA was extracted from samples by the QIAamp DNA Mini kit (QIAGEN, #51306) and the *HTT* locus amplified by PCR (6-FAM-labeled F. primer: AAGGCCTTCGAGTCCCTCAAGTCCTT; R. primer: CGGCTGAGGCAGCAGCGGCTGT). The PCR product was denatured and analyzed by capillary electrophoresis, on an Applied Bioscience 3730XL DNA Analyzer (Thermo). Chromatographs were aligned in GeneMapper v6. software (Thermo). To calculate modal CAG repeat length and instability index, GeneMapper data was exported and analyzed with a custom R script, available at https://caginstability.ml with an inclusion threshold of 20% of modal peak height and manually confirmed.

#### Microsatellite instability (MSI) analysis

DNA from ChIP samples was amplified in parallel by fluorescently labeled PCR at unstable tetranucleotide (D8S321, D20S82, D9S242, MYCL1, D20S85), dinucleotide (D2S123, D5S346, D17S250, D18S64, D18S69), mononucleotide (NR-21, NR-24, BAT-25, BAT-26, MONO-27, NR-27) and stable control pentanucleotide (Penta C and Penta D) loci. Fluorescently labeled fragments were separated by capillary electrophoresis and the repeat length of each allele determined with a custom R script, as above.

#### Transcriptome analysis

Transcriptome analysis was performed according to Sjöstedt et al. (2020). Transcriptome datasets were downloaded from The Human Protein Atlas database (https://www.proteinatlas.org/; accessed June, 2021). Data for FAN1, MLH1 and MSH3 was parsed for the cortex and striatum brain regions and graphs were generated using GraphPad Prism (v.9).

#### Mass-spectrometry

Lymphoblastoid cells, expressing endogenous levels of FAN1, and HEK293T cells transiently overexpressing myc-FAN1, were lysed 10 min on ice using PBS, 1% NP-40, Benzonase and protease inhibitors and centrifuged 5 min at 20,000 g to remove cell debris. Anti-c-*myc* magnetic beads were incubated 2 h with HEK cell lysates. A sheep FAN1 antibody (Goold et al., 2019) was incubated for 1 h with LB cell lysate and protein G magnetic beads were then added to the mix and incubated for an additional 1 h. Four washing steps were performed using lysis buffer. Crosslinking was done using 1 mM BS3 d0/d12 for 30 min at 37°C. The reaction was quenched for 20 min at 37°C using ammonium bicarbonate at a final concentration of 100 mM. Prior to digestion, beads were resuspended in a buffer containing 2 M Urea, 100 mM ammonium bicarbonate, 10 mM DTT and denatured for 20 min at room temperature under agitation (1000 rpms) (Makowski et al., 2016). Samples were then alkylated, at room temperature and in the dark, using a final concentration of 50 mM iodoacetamide for 20 min, and diluted with 50 mM ammonium bicarbonate solution to obtain a final concentration of urea below 1 M. Digestion was performed using sequencing grade trypsin overnight at 37°C. Samples were fractionated in 3 fractions using C18-SCX StageTips prepared *in-house* as previously described (Rappsilber et al., 2007) with the following concentrations of ammonium acetate: 200 mM, 1 M and 1.5 M. Prior to mass spectrometry analysis, samples were further processed using C18 StageTips.

Crosslinked peptide mixtures were resuspended in 3% acetonitrile, 0.1% formic acid and were analyzed by nano-LC-MS/MS using an Acquity M-Class system coupled to a Synapt G2Si mass spectrometer (Waters Corporation). Samples were loaded on the system and desalted by a reversed-phase Symmetry C18 trap column (180 μm internal diameter, 20 mm length, 5 μm particle size, Waters Corporation) at a flow rate of 8 μL/min for 3 min in 99% solvent A (Solvent A: MS-grade water, 0.1% formic acid – solvent B: Acetonitrile, 0.1% formic acid). Peptides were then separated using a linear gradient (0.3 μL/min, 35°C; 3%–60% solvent B over 90 min) using a BEH130 C18 nanocolumn (75 μm internal diameter, 400 mm length, 1.7 μm particle size, Waters Corporation). The mass spectrometer was operated in data-dependent acquisition mode using a mass range of 50-2000 Th for both MS and MS/MS scans and scan times of 0.2 s and 0.3 s respectively. The ten most intense precursor ions with a charge state between 3+ and 6+ were selected for fragmentation using the ‘mid’ collision energy ramp as described in James et al. (2019). Dynamic exclusion was used with a 30 s window to prevent repeated selection of peptides.

Raw mass spectrometry files were converted to MGF (Mascot Generic Format) using PLGS (v3.0.2) using slow deisotoping algorithm and automatic denoising for both MS and MS/MS data. MGF files were further converted to mzXML with MSConvert (Chambers et al., 2012) using 32-bit binary encryption.

Crosslinking identification was performed using xQuest/xProphet (Leitner et al., 2014). Searches were performed using a database containing the sequences of FAN1, MLH1, PMS2, FANCD2 and FANC1 using a search tolerance of 20 ppm. The amino acids involved in crosslinking reactions parameter was set to K, S, T, Y and N-terminal amino acid. Up to three missed cleavages were allowed, carbamidomethylation of cysteine was set as a fixed modification and oxidation of methionine was set as a variable modification. Results were validated using xProphet with a 5% FDR.

Further validation of the crosslinks was performed by extracting the highest-ranking identification from the xProphet xml output, using a modified version of Validate XL (James et al., 2019), and only considering crosslinks scoring higher than 20. For these crosslinks, the presence of light and heavy crosslinked doublets in the RAW MS files was confirmed. Automated generation of tables and MGF files was done using an *in-house* Python script to allow crosslinking map representation using xiVIEW (Mendes et al., 2019).

The mass spectrometry proteomics data have been deposited to the ProteomeXchange Consortium via the PRIDE (Perez-Riverol et al., 2019) partner repository with the dataset identifier PXD023221 (Username: reviewer_pxd023221@ebi.ac.uk - Password: lSFFWYxO). Code used for data processing is available at https://github.com/tmenneteau/xq-processing.

### Quantification and statistical analysis

CAG expansion time courses were analyzed by linear regression in GraphPad Prism (v9) and slopes statistically compared by one-way ANOVA. Multiple comparisons were corrected for with a False Discovery Rate (FDR) of 5%. Area under curve (AUC) data were compared by a one-way ANOVA with an FDR correction of 5%. Significance was defined using FDR-corrected p values. Data between two groups were analyzed by independent-samples t tests. ^∗^p < 0.05, ^∗∗^ p < 0.01, ^∗∗∗^p < 0.001, ns = non-significant. The Brown-Forsythe test was routinely used to check for homogeneity of variance. All statistical information can be found within figure legends.

For conservation analysis, the human FAN1 sequence was aligned in HomoloGene (NCBI) with common model species and visualized with SnapGene software.

## Data Availability

• Mass spectrometry proteomics data are deposited at ProteomeXchange: PXD023221 (Username: reviewer_pxd023221@ebi.ac.uk - Password: lSFFWYxO). • Fragment analysis software is available at https://caginstability.ml. • Any additional information required to reanalyze the data reported in this work is available from the Lead Contact upon request.

## Supplemental information

Document S1. Figures S1–S3 and Table S1

Document S2. Article plus supplemental information
