# Supplementary material for: FAN1 controls mismatch repair complex assembly via MLH1 retention to stabilize CAG repeat expansion in Huntington’s disease
Source: Cell Rep. 2021 Aug 31;36(9):109649. doi: 10.1016/j.celrep.2021.109649 (PMC8424649; doi:10.1016/j.celrep.2021.109649)
Supplement: Document S1. Figures S1–S3 and Table S1 [file mmc1.pdf]

Cell Reports, Volume *36*

Supplemental information

FAN1 controls mismatch repair complex assembly via MLH1 retention to stabilize CAG repeat expansion in Huntington's disease

Robert Goold, Joseph Hamilton, Thomas Menneteau, Michael Flower, Emma L. Bunting, Sarah G. Aldous, Antonio Porro, José R. Vicente, Nicholas D. Allen, Hilary Wilkinson, Gillian P. Bates, Alessandro A. Sartori, Konstantinos Thalassinos, Gabriel Balmus, and Sarah J. Tabrizi

Supplementary figures

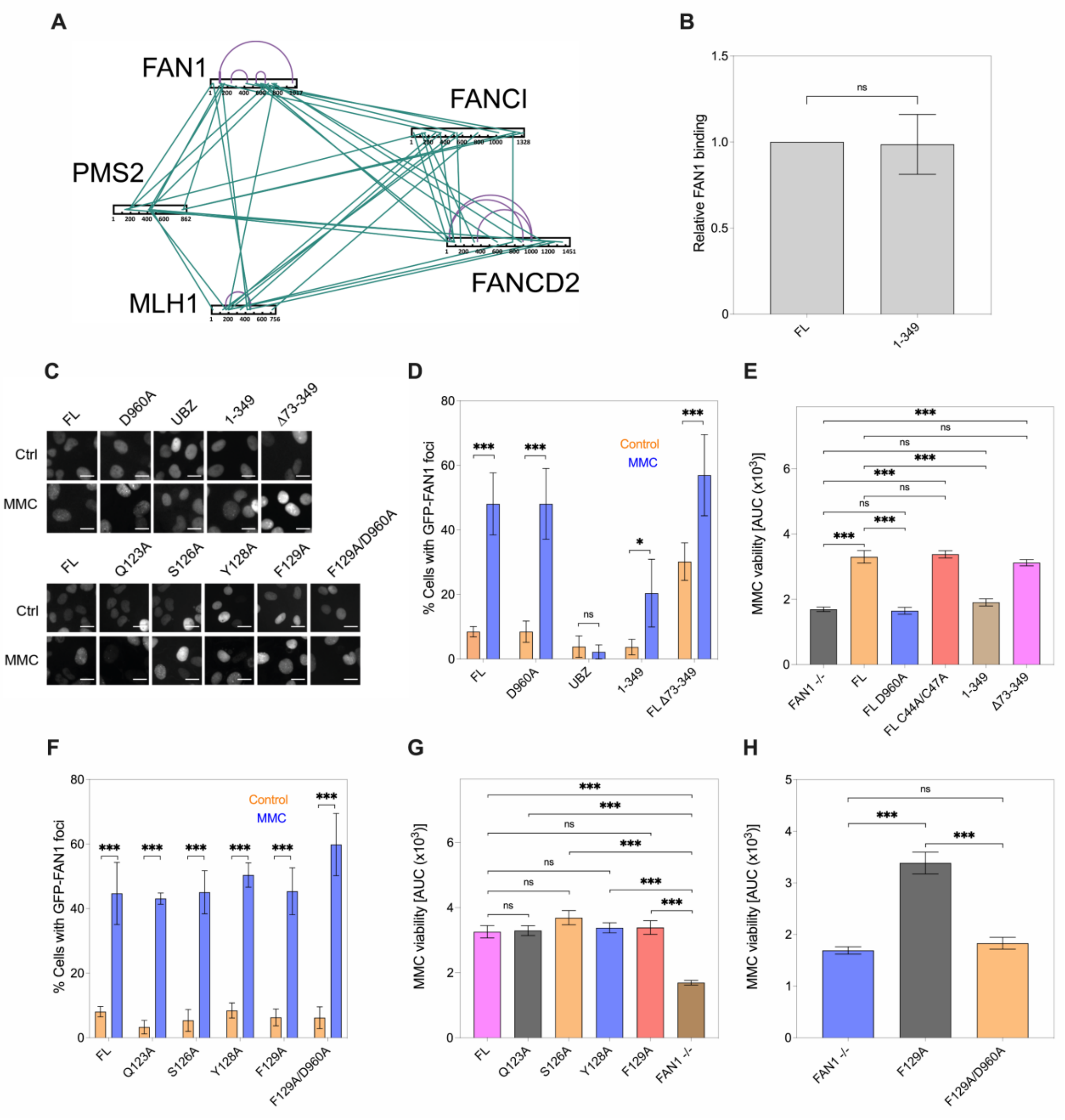

Figure S1. FAN1-MLH1 interactions and functional characterisation of U2OS FAN1 constructs

(A) Identified crosslinks between FAN1, MLH1, PMS2, FANCD2 and FANCI. The crosslink map was generated using xiVIEW. Inter-protein crosslinks are shown in green and intra-protein crosslinks are shown in purple. (Related to Figure 1E, Table S1)

(B) Relative FAN1 binding in MLH1 IP fractions between FL FAN1 and FAN1 1-349 genotypes. (mean ± SD, n=3 biological replicates, $t(4)=0.077$, $p=0.943$ by independent-samples t-test) ns = non-significant (Related to Figure 1G)

(C) GFP live cell imaging of U2OS cells expressing the indicated FAN1-GFP fusion constructs with quantification (D,F). Note the nuclear localization and formation of DNA repair foci in response to MMC in all lines but UBZ cells (mean ± SEM, n=3 biological replicates , Scale bar= 50uM, [C] WT: $t(20)=6.54$, $p<0.001$, D960A: $t(20)=6.54$, $p<0.001$, UBZ: $t(20)=0.27$, $p=0.789$, 1-349: $t(20)=2.76$, $p=0.12$, $WT^{\Delta 73\text{-}349}$: $t(20)=4.42$, $p<0.001$, Q123A; [D] WT:$t(20)=9.21$, $p<0.001$, Q123A: $t(20)=10$, $p<0.001$, S126A: $t(20)=9.99$, $p<0.001$, Y128A: $t(20)=10.55$, $p<0.001$, F129A: $t(20)=9.83$, $p<0.001$ by independent-samples t-tests) *$p<0.05$, ***$p<0.001$, ns = non-significant (Related to Figure 1)

(E) Quantification of MMC viability curves in U2OS cells expressing FAN1, showing lower viability when FAN1 lacks an intact nuclease domain (mean ± SEM, n=5-8 biological replicates, $F(5,204)=36.21$, $p<0.001$ by one-way ANOVA with FDR correction of 5%). ***$p<0.001$, ns = non-significant (Related to Figure 1I)

(G) Quantification of MMC viability curves in U2OS cells expressing FAN1 SPYF mutants, showing no significant difference in viability to cells expressing $FAN1^{FL}$ (mean ± SEM, n=6-8 biological replicates, $F(5,257)=14.25$, $p<0.001$ by one-way ANOVA with FDR correction of 5%). ***$p<0.001$, ns = non-significant (Related to Figure 2F)

(H) Quantification of MMC viability curves in U2OS cells expressing $FAN1^{F129A/D960A}$ mutant, showing no significant difference in viability to $FAN1^{-/-}$ cells (mean ± SEM, n=6-7 biological replicates, $F(2,112)=42.1$, $p<0.001$ by one-way ANOVA with FDR correction of 5%). ***$p<0.001$, ns = non-significant (Related to Figure 3D)

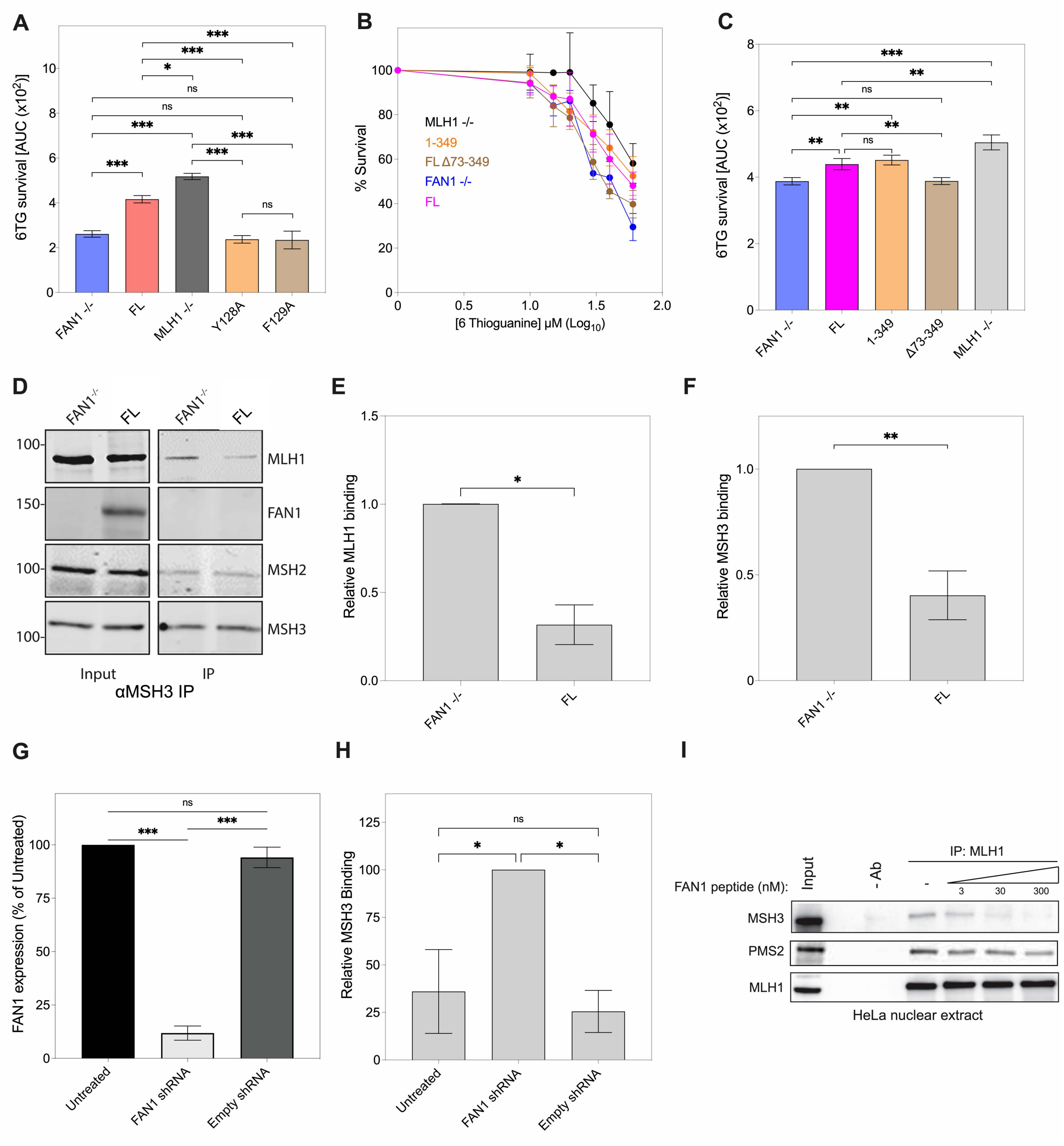

Figure S2. FAN1 regulates mismatch repair activity and MSH3-MLH1 complex formation

(A) Quantification of 6TG viability curves in U2OS cells expressing FAN1 SPYF mutants (mean ± SD). Note that FAN1 SPYF mutants have decreased 6TG resistance, similar to FAN1$^{-/-}$, whereas FAN1^{FL} 6TG resistance approaches but does not reach MLH1$^{-/-}$ levels. (mean ± SEM, n=5 biological replicates, $F(4,106)=23.09$, $p<0.001$ by one-way ANOVA with FDR correction of 5%) . * p<0.05, ***p<0.001, ns = non-significant (Related to Figure 4D)

(B) 6TG viability curves in U2OS cells expressing FAN1 deletion constructs (mean ± SD) with quantification (C). Note that FAN1 lacking amino acids 73-349 has decreased 6TG resistance,

similar to $FAN1^{-/-}$, whereas $FAN1^{1-349}$ maintains 6TG resistance but does not reach $MLH1^{-/-}$ levels. (mean ± SEM, n=5 biological replicates, $F(3,104)=13.49$, $p<0.001$ by one-way ANOVA with FDR correction of 5%). ** $p<0.01$, ***$p<0.001$, ns = non-significant (Related to Figure 4)

(D) Co-IP extracts using αMSH3 antibody in U2OS cells showing reduced MLH1 binding when FAN1 is expressed. Note the absence of FAN1 in the MSH3 IP fraction and the stable levels of MSH2 between lines (n=2 biological replicates). (Related to Figure 4)

(E) Relative MLH1 binding in MSH3 IP fractions between $FAN1^{FL}$ and $FAN1^{-/-}$ genotypes. (mean ± SD, n=2 biological replicates, $t(2)=6.1$, $p=0.026$ by independent-samples t-test) ns = non-significant (Related to Figure 4)

(F) Quantification of Co-IP of MLH1 and binding partners from $FAN1^{-/-}$ and $FAN1^{WT}$ cells. Note FAN1 expression reduces MSH3 levels in MLH1 IP fractions (mean ± SEM, n=4 biological replicates, $t(6)=5.2$, $p=0.001$ by independent-samples t-test). **$p<0.01$ (Related to Figure 4E)

(G) Stable incorporation of shRNA sequences targeting FAN1 reduce protein levels relative to Untreated or Empty shRNA vector-treated controls (mean ± SEM, n=6 biological replicates, F(2,15=212.4, $p<0.001$ by one-way ANOVA with FDR correction of 5%). ***$p<0.001$, ns = non-significant (Related to Figure 4F)

(H) Quantification of Co-IP of MLH1 and binding partners from control (Untreated and FAN1 Empty vector) and FAN1 knock down 125 CAG HD-MSNs. Note FAN1 knock down increases MSH3 levels in MLH1 IP fractions (mean ± SEM, n=3 biological replicates, $F(2,6)=8.014$, $p=0.02$ by one-way ANOVA with FDR correction of 5%). *$p<0.05$, ns = non-significant (Related to Figure 4F)

(I) Peptide competition assay in HeLa nuclear extracts showing FAN1 60-mer peptides reduce endogenous MLH1-MSH3 interactions in a dose-dependent manner (n=3 biological replicates). (Related to Figure 4H)

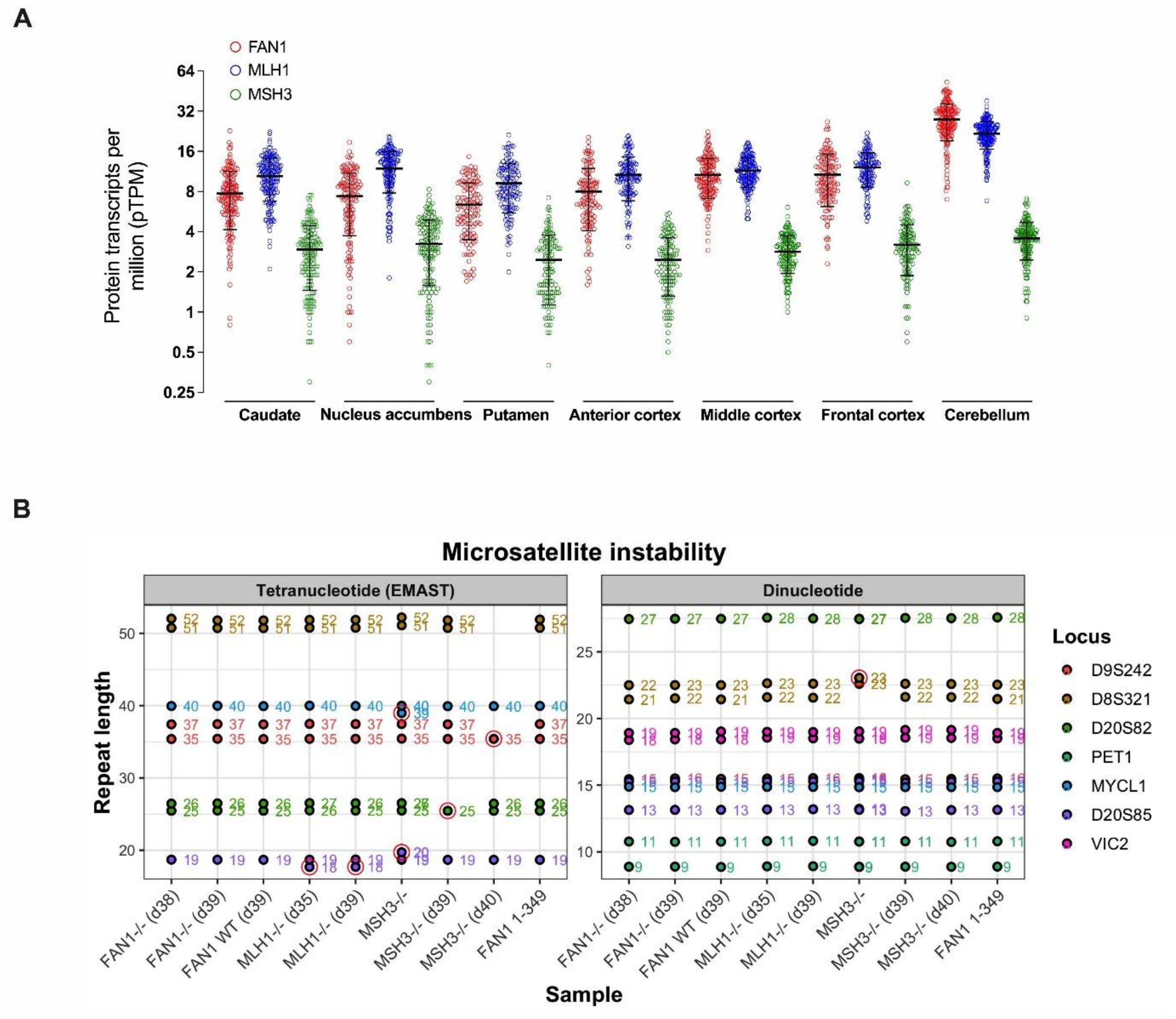

Figure S3. DNA repair gene expression in human tissues and microsatellite instability within U2OS genotypes

(A) Column scatter plot showing the relative mRNA expression level of FAN1, MLH1 and MSH3 in the striatum and cortex (pTPM, protein transcripts per million). Data was collected from human non-diseased brain tissue samples (Striatum: caudate, N=160; nucleus accumbens, N=147; putamen, N=124. Cortex: anterior, N=121; middle, N=158; frontal, N=129). Error bars denote standard deviation. (Related to Figure 4)

(B) MLH1 and MSH3 knockout causes microsatellite instability at tetra- and dinucleotide repeats. Repeat length for both alleles are plotted for U2OS cells of the given genotypes. Each point represents an allele, and colours represent genomic loci, including tetranucleotide

(D8S321, D20S82, D9S242, MYCL1, D20S85), dinucleotide (D2S123, D5S346, D17S250, D18S64, D18S69) and stable control pentanucleotide (Penta C and Penta D; data not shown) loci. Those showing microsatellite instability (MSI) are circled red. MLH1 and MSH3 knockout induces repeat contraction or expansion at tetranucleotide and dinucleotide loci, including D20S85, MYCL1, D20S82, D9S242 and D17S250 (n=3 biological replicates, n=3-6 technical replicates). (Related to Figure 4)

Peptide sequence 1	Peptide sequence 2	Protein 1 Uniprot entry name	Protein 2 Uniprot entry name	xQuest score	Crosslink position in peptide 1	Crosslink position in peptide 2	Crosslink position in protein 1	Crosslink position in protein 2
NIGFSHLQQR	TRKQPLSK	FACD2_HUMAN	FACD2_HUMAN	34.37	5	3	1002	25
KTLELLVCR	TPYPRPR	FACD2_HUMAN	PMS2_HUMAN	31.56	2	3	1363	149
LQEFLQTLR	MVSKRR	FANCI_HUMAN	FACD2_HUMAN	30.99	7	3	23	3
TNLTPGQSDSAKR	KSPPPK	FAN1_HUMAN	FAN1_HUMAN	30.8	10	2	116	102
RLDETVVNR	RSPLGQK	MLH1_HUMAN	PMS2_HUMAN	30.18	5	2	14	445
NPSEEYGK	LDYELGR	MLH1_HUMAN	FANCI_HUMAN	29.55	3	3	170	283
GGLPDLVVWNSQSR	KSPPPK	FAN1_HUMAN	FAN1_HUMAN	29.51	13	2	968	102
HVTITGRLCPQR	KDVSISR	FAN1_HUMAN	PMS2_HUMAN	29.09	3	4	781	416
RHPSYPK	TGEEKK	FACD2_HUMAN	PMS2_HUMAN	29.00	4	5	88	412
YSVHNAGISFSVKKQGETVADVR	LESMSAK	MLH1_HUMAN	FACD2_HUMAN	28.07	14	3	196	773
NSECDPTPSHR	QPLSKK	FACD2_HUMAN	FACD2_HUMAN	27.77	7	5	896	30
QGETVADVR	IASLAR	MLH1_HUMAN	FACD2_HUMAN	27.74	4	3	200	1157
LSLYQRAVR	KQKTDGSK	FAN1_HUMAN	FACD2_HUMAN	27.24	4	7	747	877
RAKALAGQSVRICK	VSMSMR	FAN1_HUMAN	FANCI_HUMAN	26.88	3	4	528	512
YEKTISEAWIK	VPFLKNK	FACD2_HUMAN	FACD2_HUMAN	26.86	3	5	358	992
CLSQQADVR	ASYSDGK	FANCI_HUMAN	MLH1_HUMAN	26.51	3	4	596	131
TNLTPGQSDSAKR	TSVVKSFK	FAN1_HUMAN	FANCI_HUMAN	26.02	4	2	110	333
LLYWNMAVR	LREAFSLR	FACD2_HUMAN	PMS2_HUMAN	25.04	3	6	1267	425
VGTRLMFDHNGKIIQK	QLINTLCSGR	PMS2_HUMAN	FANCI_HUMAN	24.13	3	5	133	163
ALAGQSVRICKGPR	NHPSLR	FAN1_HUMAN	FAN1_HUMAN	23.68	11	4	539	646
LQEFLQTLR	MVSKRR	FANCI_HUMAN	FACD2_HUMAN	23.05	7	4	23	4
TGEEKKDVSISR	KYVKAKK	PMS2_HUMAN	FAN1_HUMAN	22.95	9	4	416	165
SFKDLQLLQGSK	LLGSNSSR	FANCI_HUMAN	MLH1_HUMAN	21.6	11	7	347	340
ALKNPSEEYGK	GTSEMSEKR	MLH1_HUMAN	MLH1_HUMAN	21.33	3	8	167	461
LQASQVKLKSKGR	KENLAYGK	FACD2_HUMAN	FANCI_HUMAN	20.98	7	6	322	146
KQGETVADVR	LRESPSCKK	MLH1_HUMAN	FAN1_HUMAN	20.96	5	8	200	760
ALKNPSEEYGK	GTSEMSEKR	MLH1_HUMAN	MLH1_HUMAN	20.61	3	3	167	456
ALAGQSVRICKGPR	LFQTLRR	FAN1_HUMAN	FACD2_HUMAN	31.67	11	4	539	82
LLMVILEKSTASAQNK	LEPTIK	FACD2_HUMAN	FAN1_HUMAN	29.87	12	4	1148	725
SNDVVCK	TDISSGR	FAN1_HUMAN	MLH1_HUMAN	29.12	1	4	131	420
TLPNASTVDNIR	VSMSMR	MLH1_HUMAN	FANCI_HUMAN	28.58	1	4	206	512
EVKQKISPYFK	SPLGQK	FAN1_HUMAN	PMS2_HUMAN	28.53	9	1	128	445
KDVSISRLR	NPSEEYGK	PMS2_HUMAN	MLH1_HUMAN	27.93	6	6	418	173
SLMNLLFSLHVSYK	KTHIFQDR	FANCI_HUMAN	FAN1_HUMAN	27.8	1	2	1027	593

Peptide sequence 1	Peptide sequence 2	Protein 1 Uniprot entry name	Protein 2 Uniprot entry name	xQuest score	Crosslink position in peptide 1	Crosslink position in peptide 2	Crosslink position in protein 1	Crosslink position in protein 2
SVMIGTALNTSEMK	VICLGSLASKLSR	PMS2_HUMAN	FAN1_HUMAN	27.55	10	10	824	158
NIKKEYAK	RSLSISK	PMS2_HUMAN	FAN1_HUMAN	27.29	6	6	181	20
KSKVNLMQHMKLSTSR	LYVRLFQR	FANCI_HUMAN	FAN1_HUMAN	26.91	11	2	1280	418
WTVEHIVYKALR	LSDILNEK	MLH1_HUMAN	FANCI_HUMAN	26.42	2	2	715	782
LFQTLRRHPSYPK	EGSLVNGK	FACD2_HUMAN	FANCI_HUMAN	25.88	10	3	88	120
KASNSIISCFNNAPPAK	GTSEMSEK	FAN1_HUMAN	MLH1_HUMAN	25.12	8	2	32	455
LLYWNMAVR	TDISSGR	FACD2_HUMAN	MLH1_HUMAN	25.09	3	5	1267	421
TSVVKSFK	KLFQTLR	FANCI_HUMAN	FACD2_HUMAN	24.38	5	5	336	82
KTHIFQDR	MVSKRR	FAN1_HUMAN	FACD2_HUMAN	23.79	2	3	593	3
KSAVAGFLLLLK	LPEYFFENK	FANCI_HUMAN	FACD2_HUMAN	23.65	2	4	535	160
KTHIFQDR	MVSKRR	FAN1_HUMAN	FACD2_HUMAN	22.83	2	4	593	4
SESPSLTQER	KTHIFQDR	FACD2_HUMAN	FAN1_HUMAN	22.57	3	2	592	593
FVEILQRLHMYEEAVR	NIKKEYAK	FAN1_HUMAN	PMS2_HUMAN	22.51	11	4	683	179
QMFASRACR	EPAKKK	PMS2_HUMAN	FANCI_HUMAN	22.31	5	5	809	1324
LSWIKMTK	ATRECEK	FAN1_HUMAN	FAN1_HUMAN	21.11	7	2	432	245
LESMSAK	NMEKLVK	FACD2_HUMAN	FANCI_HUMAN	20.69	5	4	775	1196
QMFASRACRK	SLNYTGEK	PMS2_HUMAN	FANCI_HUMAN	20.05	5	1	809	1222

Table S1: List of the crosslinks identified between FAN1, MLH1, PMS2, FANCD2 and FANCI. (Related to Figure 1D and Figure S1A)

Peptide sequences pairs involved in crosslinks are listed with the xQuest score and the position of the crosslink in the peptide and the protein.
